# The complete chloroplast genome of *Chimonobambusa purpurea* (Bambusatae)

**DOI:** 10.1080/23802359.2021.1882897

**Published:** 2021-03-10

**Authors:** Wei Liu, Jun Zhang, Yun-Ke Liu, Qian-Ying Huang, Qian-Gang Xiao, Bao-Xin Wang

**Affiliations:** a Chengdu Academy of Agricultural and Forestry Sciences, Chengdu, Sichuan, PR China; b Key Laboratory of Ecological Forestry Engineering of Sichuan Province, College of Forestry, Sichuan Agricultural University, Chengdu, Sichuan, PR China

**Keywords:** *Chimonobambusa purpurea*, chloroplast genome, phylogenetic analysis

## Abstract

*Chimonobambusa purpurea* is one of the important bamboo species in southwest of China. We studied the complete chloroplast (cp) genome of *C. purpurea* in this study. The cp genome of *C. purpurea* (GenBank accession: MW030500) was 139,574 bp in length, including a large single-copy (LSC) region of 83,171 bp, a small single-copy (SSC) region of 12,811 bp, and a pair of inverted repeated (IR) regions of 21,796 bp. And the genome contained 133 genes, including 86 protein-coding genes, 39 tRNA genes, and 8 rRNA genes. Based on 30 cp genomes, we used the phylogenetic analysis to build phylogenetic tree, indicating that *C. purpurea* is closely related to *C. tumidissinoda.*

*Chimonobambusa purpurea* is one of the important bamboo species in southwest of China. *C. purpurea* is mainly distributed in Shaanxi, Hubei, and Sichuan in China (Li and Stapleton 2006). The bamboo poles of *C. purpurea* can be used as raw materials for papermaking and the bamboo shoots is edible, which has potential commercial value. In this study, we have studied and characterized the chloroplast genome of *C. purpurea*, and provide genomic and genetic resources for further research.

Genomic DNA was extracted from fresh leaves of an individual of *C. purpurea*. The specimens of *C. purpurea* were collected from Chongzhou Country, Chengdu City, Sichuan Province, China (N30°45′27″, E103°25′14″) on 2 June 2020. The specimens were deposited in the herbarium room of Chengdu Academy of Agricultural and Forestry Sciences (http://www.cdnky.com/, Bao-Xin Wang and wangbaoxin0101@163.com) under the voucher number LW20200602-01. First, a whole-genome shotgun library with an insert size of 400 bp was prepared, and then the library was sequenced by the Illumina NovaSeq platform and thus generating 150 bp paired-end reads. Ten million high-quality reads were mapped to the published *Bambusa emeiensis* chloroplast genomes as references using mummer version 3.1 (Kurtz et al. 2004). Second, we using A5-miseq v20150522 (Coil et al. 2015) and SPAdes version 3.9.0 to assemble these reads into complete chloroplast genomes (Bankevich et al. 2012). The assembled chloroplast genome sequence was annotated using the online Geseq web server (https://chlorobox.mpimp-golm.mpg.de/geseq.html). Follow the default parameters.

The complete plastid genome sequence of *C. purpurea* (GenBank accession: MW030500) was 139,574 bp in length, including a large single-copy (LSC) region of 83,171 bp, a small single-copy (SSC) region of 12,811 bp, and a pair of inverted repeated (IR) regions of 21,796 bp. The complete chloroplast genome contained 133 genes, including 86 protein-coding genes, 39 tRNA genes, and 8 rRNA genes. The complete genome GC content was 38.90%, and the corresponding values of the LSC, SSC, and IR were 36.99%, 33.23%, and 44.23%, respectively.

The maximum-likelihood phylogenetic tree was constructed based on 30 complete chloroplast genomes of Bambusoideae species, and *Phaenosperma globosum* as outgroup. The sequences were aligned by MAFFT version 7.037 (Katoh and Standley 2013), and the phylogenetic tree constructed by IQ-TREE Multicore version 1.6.12 (Gao et al. 2018). The result indicated that *C. purpurea* has a close relationship with *C. tumidissinoda* (Figure 1).

**Figure 1.**
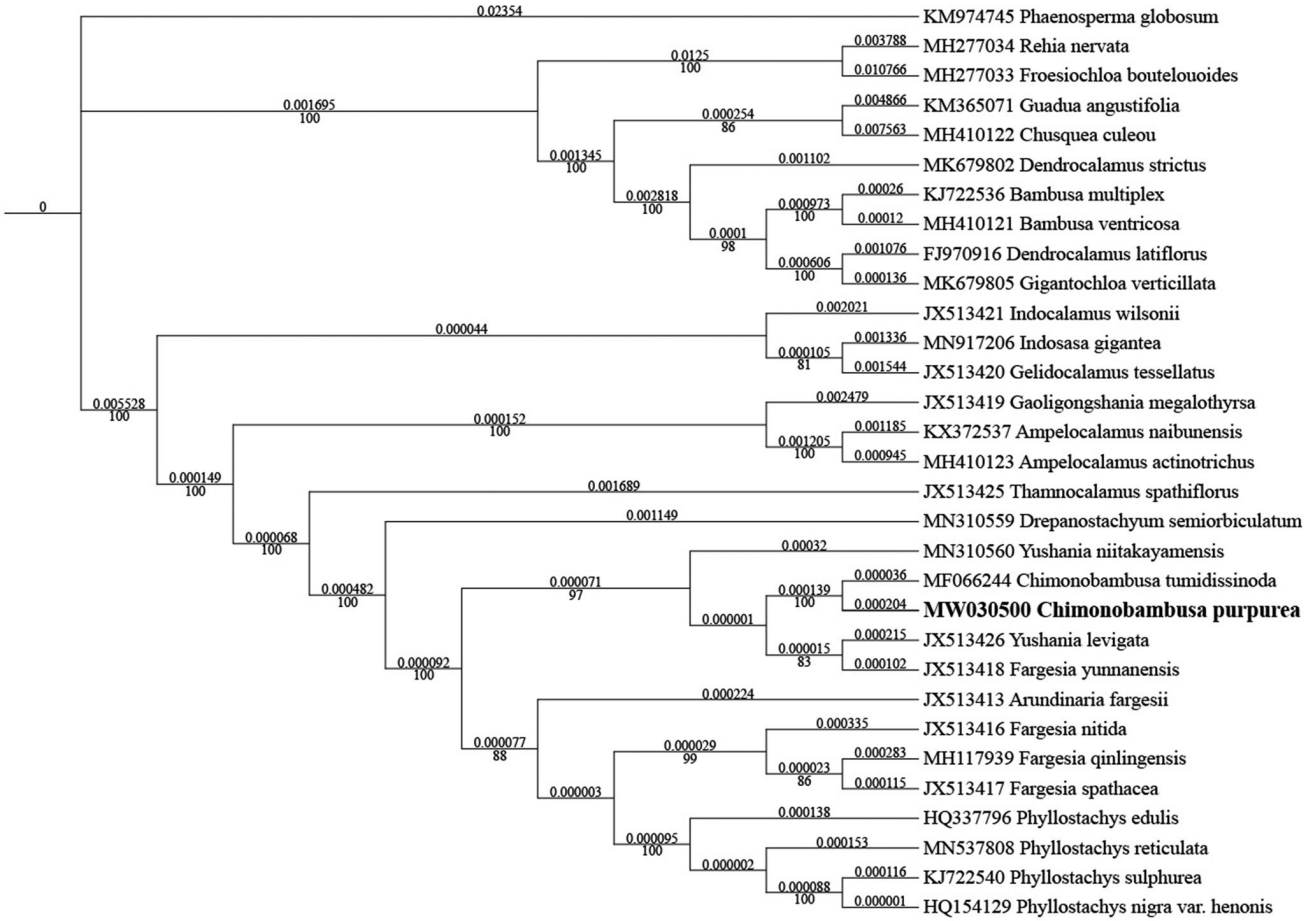
Maximum-likelihood phylogenetic analysis of 30 species of Bambusoideae and *Phaenosperma globosum* as outgroup based on plastid genome sequences by IQ-TREE multicore version 1.6.12 under GTR + R6 model for 5000 ultrafast bootstraps. Branch lengths (above) and bootstrap values (below) were indicated around nodes. GeneBank accession numbers of each species were listed in the tree.

## Data Availability

The genome sequence data that support the findings of this study are openly available in GenBank of NCBI at (https://www.ncbi.nlm.nih.gov/) under the accession no. MW030500. The associated BioProject, SRA, and BioSample numbers are PRJNA684786, SRR13258662, and SAMN17069327, respectively.
